# Sex chromosome diversity in Armenian toad grasshoppers (Orthoptera, Acridoidea, Pamphagidae)

**DOI:** 10.3897/CompCytogen.v10i1.6407

**Published:** 2016-01-22

**Authors:** Alexander G. Bugrov, Ilyas E. Jetybayev, Gayane H. Karagyan, Nicolay B. Rubtsov

**Affiliations:** 1 Institute of Systematics and Ecology of Animals, Russian Academy of Sciences, Siberian Branch, Frunze St. 11, 630091 Novosibirsk, Russia; 2 Novosibirsk State University, Pirogov St., 2, 630090 Novosibirsk, Russia; 3 Institute of Cytology and Genetics, Russian Academy of Sciences, Siberian Branch, Pr. Lavrentjeva, 630090 Novosibirsk, Russia; 4 Scientific Center of Zoology and Hydroecology NAS RA, P. Sevak 7, 0014 Yerevan, Armenia

**Keywords:** Pamphagidae grasshoppers, karyotype, autosome, neo sex chromosome evolution, neo-X, neo-Y chromosomes, FISH analysis, rDNA and telomeric repeats

## Abstract

Although previous cytogenetic analysis of Pamphagidae grasshoppers pointed to considerable karyotype uniformity among most of the species in the family, our study of species from Armenia has discovered other, previously unknown karyotypes, differing from the standard for Pamphagidae mainly in having unusual sets of sex chromosomes. *Asiotmethis turritus* (Fischer von Waldheim, 1833), *Paranocaracris rubripes* (Fischer von Waldheim, 1846), and *Nocaracris cyanipes* (Fischer von Waldheim, 1846) were found to have the karyotype 2n♂=16+neo-XY and 2n♀=16+neo-XX, the neo-X chromosome being the result of centromeric fusion of an ancient acrocentric X chromosome and a large acrocentric autosome. The karyotype of *Paranothrotes opacus* (Brunner von Wattenwyl, 1882) was found to be 2n♂=14+X_1_X_2_Y and 2n♀=14+X_1_X_1_X_2_X_2._, the result of an additional chromosome rearrangement involving translocation of the neo-Y and another large autosome. Furthermore, evolution of the sex chromosomes in these species has involved different variants of heterochromatinization and miniaturization of the neo-Y. The karyotype of *Eremopeza festiva* (Saussure, 1884), in turn, appeared to have the standard sex determination system described earlier for Pamphagidae grasshoppers, 2n♂=18+X0 and 2n♀=18+XX, but all the chromosomes of this species were found to have small second C-positive arms. Using fluorescent *in situ* hybridization (FISH) with 18S rDNA and telomeric (TTAGG)_n_ DNA repeats to yield new data on the structural organization of chromosomes in the species studied, we found that for most of them, clusters of repeats homologous to 18S rDNA localize on two, three or four pairs of autosomes and on the X. In *Eremopeza festiva*, however, FISH with labelled 18S rDNA painted C-positive regions of all autosomes and the X chromosome; clusters of telomeric repeats localized primarily on the ends of the chromosome arms. Overall, we conclude that the different stages of neo-Y degradation revealed in the Pamphagidae species studied make the family a very promising and useful model for studying sex chromosome evolution.

fluorescent *in situ* hybridization

## Introduction

About 600 species of Pamphagidae, or “toad grasshoppers” as they are commonly known, are distributed in the desert and mountainous landscapes of Africa, Central Asia, and the Western and Eastern Mediterranean (Uvarov 1966, Massa 2013), including some species found in Armenia (Avakian 1968). To date, the family Pamphagidae remains one of most poorly karyotyped groups among the grasshoppers. One reason for this lies in the very low densities of the toad grasshopper populations. Until recently, exceptional karyotypic conservatism was considered a prominent feature of this family. Diploid sets of chromosomes in these grasshoppers consisted of 19 (♂) and 20 (♀) acrocentric chromosomes; the described sex determination were XO♂/XX♀ (White 1973, Hewitt 1979, Camacho et al. 1981, Santos et al. 1983, Cabrero et al. 1985, Fu Peng 1989, Mansueto and Vitturi 1989, Vitturi et al. 1993, Warchałowska-Śliwa et al. 1994).

However, karyotyping of some Pamphagidae species from Central Asia and Bulgaria forced researchers to reconsider this notion of a uniform karyotype structure within the family. Reciprocal translocation between an ancient acrocentric X chromosome and one of the acrocentric autosomes was discovered to have taken place in some pamphagid species (Bugrov 1986, Bugrov and Warchałowska-Śliwa 1997, Bugrov and Grozeva 1998, Li et al. 2005), leading to the formation of a large, biarmed neo-X chromosome. Following the nomenclature of White (1940), the arm of the neo-X chromosome derived from the ancient acrocentric X chromosome is denoted as an XL arm, the autosome part of the neo-X as an XR arm. In males, the autosome homologous to the XR arm is referred to as the neo-Y chromosome.

This article reports the results of our comparative analysis of the karyotypes of Armenian Pamphagidae grasshoppers and our study of the structural organization of their chromosomes, including the distribution of clusters of telomeric repeats and repetitive DNA homologous to 18S rDNA in the chromosomes of the species studied.

## Material and methods

### Material collection and karyotype analyses

Males of *Eremopeza festiva* (Saussure, 1884) (n=19), *Asiotmethis turritus* (Fischer von Waldheim, 1833) (subfamily Thrinchinae) (n=5); *Nocaracris cyanipes* (Fischer von Waldheim, 1846) (n=9), *Paranocaracris rubripes* (Fischer von Waldheim, 1846) (n=6), and *Paranothrotes opacus* (Brunner von Wattenwyl, 1882) (n=3) (subfamily Pamphaginae, tribe Nocarodeini) were collected in the early summer season of 2013 in mountain and desert landscapes in Armenia.

After 0.1% colchicine solution was injected into males for 1.5–2.0 hours, the tests testes were dissected and placed into 0.1% solution of sodium citrate for 15 minutes, fixed in 3:1 ethanol:glacial acetic acid for 15 minutes and kept in 70% ethanol. Air-dried chromosome preparations were made by the standard squashing technique (Darlington and La Cour 1976).

Three females of *Paranocaracris rubripes* were kept in cages with moisturized sand to obtain egg pods. Each portion of eggs was stored in a separate Petri dish with moist sand and placed in an incubator. After 15–20 days of incubation at room temperature, the eggs were placed into a solution of 0.05% colchicine in 0.9% sodium citrate and the tops of the nonmicropylar ends were removed. They were then incubated at 30 °C for 1.5–2 h. The eggs were fixed in 3:1 ethanol:acetic acid for 15 minutes and embryos were dissected out of the eggs and transferred into distilled water for 15–20 min at room temperature. Air-dried preparations were made on pre-cleaned slides by macerating the embryos in a drop of 60% acetic acid.

C-banding of the chromosome preparations was performed according to Sumner’s protocol (1972). Slides were treated with 0.2 N HCL for 15–20 min, then incubated in the saturated solution of Ba(OH)_2_ at 61 °C for 3–5 min, rinsed in water, and placed into 2xSSC at 61 °C for 60 min. After being rinsed in water the slides were stained with 2% Giemsa solution.

### Fluorescence *in situ* hybridization


 Fluorescence *in situ* hybridization (FISH) on mitotic and meiotic chromosomes was carried out as described in previous studies (Rubtsov et al. 2000, Rubtsov et al. 2002). Clusters of repetitive DNA homologous to 18S rDNA in metaphase chromosomes were visualized by FISH with a fragment of human 18S rDNA (Malygin 1992) labelled with digoxygenin or biotin. DNA labelling was performed as described in Rubtsov et al. (2000). The DNA probe for detection of telomeric repeats (TTAGG)_n_ in metaphase chromosomes was generated with non-template PCR (Ijdo et al. 1991) with 5´-TAACCTAACCTAACCTAACC-3´ and 5´-TTAGGTTAGGTTAGGTTAGG-3´ primers according to standard protocol (Sahara et al. 1999).

Visualization of hybridized DNA labelled with digoxygenin or biotin was performed with antidigoxigenin sheep antibodies conjugated with Rhodamine or with avidin-FITC conjugates, respectively.

Chromosome counterstaining was preformed after FISH with 4´,6-diamidino-2-phenylindole (DAPI) using Vectashield antifade containing 200ng/ml DAPI. Vectashield antifade was put under glass cover, which was then sealed with rubber cement.

### Microscope analysis

Microscopic analysis was carried out at the Center for Microscopy of Biological Subjects (Institute of Cytology and Genetics, Novosibirsk, Russia). Chromosomes were studied with an AxioImager M1 (Zeiss) fluorescence microscope equipped with filter sets #49, #46HE, #43HE (Zeiss) and a ProgRes MF (MetaSystems) CCD camera. The ISIS5 software package (MetaSystems GmbH, Germany) was used for image capture and analysis.

### Chromosome nomenclature

The nomenclature suggested for Pamphagidae grasshoppers (Camacho et al. 1981) was used in the description of chromosomes and karyotypes.

## Results

**Figure 1.**
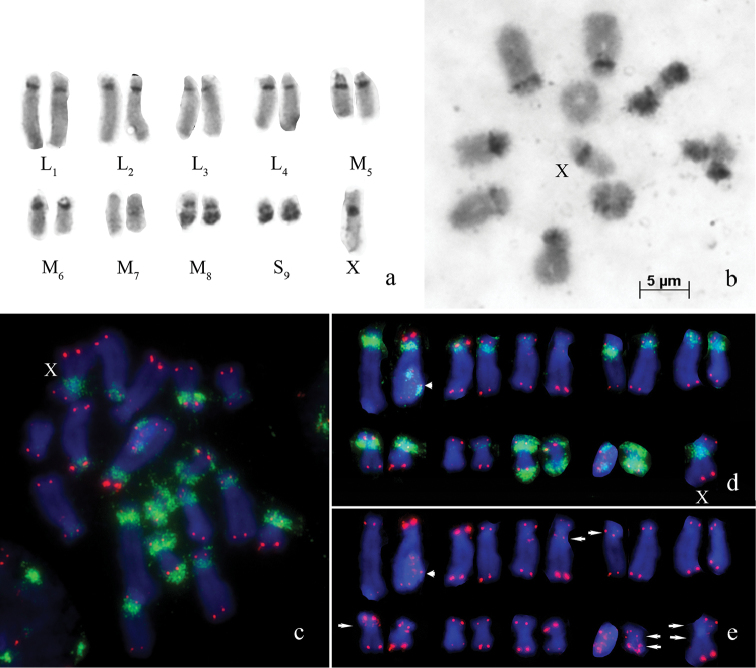
*Eremopeza festiva* – **a** C-banded spermatogonial metaphase chromosomes **b** C-banded meiotic metaphase I **c, d** Fluorescence *in situ* hybridization (FISH) with 18S rDNA probe (green) and a telomeric repeat probe (red) with – spermatogonial metaphase chromosomes, arrowhead indicate chromosome S_9_ overlapping on chromosome L_2_ **e** Fluorescence *in situ* hybridization (FISH) with telomeric repeats (red) on the same chromosome plate without green channel, arrows indicate interstitial telomeric sites. Bar: 5 µm.

**Karyotype description of *Eremopeza festiva* (Saussure, 1884).** The karyotype of *Eremopeza festiva* consisted of biarmed chromosomes, 2n♂=19, (18AA+X). The autosomes fell into three groups based on size: 4 long (L_1_–L_4_), 4 medium (M_5_–M_8_), and 1 short (S_9_). The size of the X chromosome is approximately similar to the L_2_ pair. Large chromosomes and the largest medium-sized pair (L_1_-L_4_ and M_5_) are subacrocentric. The M_6_, M_7_, M_8_, S_9_ pairs and the X chromosome are submetacentrics. Pericentric C-blocks appeared in all chromosomes of the complement except M_7_. The size of the C-blocks varied for different chromosome pairs. In the smallest chromosomes of the set (M_8_ and S_9_), distal C-positive blocks were observed on their long arms (Fig. 1a). The short chromosome arms in the autosomes are C-negative. In the prophase of meiosis, the long autosomes form 2–3 chiasmata, the medium-sized ones form 1–2, and the short autosome forms only one chiasma (Fig. 1b).

The telomeric DNA probe hybridized on the termini of all chromosomes. FISH signals of the telomeric DNA probe differed in size and intensity on chromosome arms (L_1_, L_3_, M_7_, M_8_, X), between homologous chromosomes (L_1_, L_3_, M_7_) (Fig. 1c, d). Besides telomeric location, polymorphic interstitial telomeric sequences (ITSs) were discovered in pericentric regions of L_3_, L_4_, M_6_, S_9_ and the X chromosome (arrows on Fig. 1e).

FISH with 18S rDNA probe returned a signal for the C-positive regions on almost all autosomes and the X chromosome. The intensity of hybridization signal varied from very intense in L_1_, L_4_, M_6_, M_8_, S_9_, to very weak in L_3_ and M_7_ chromosome pairs. (Fig. 1c, d). The FISH signal was also observed in the C-negative short arms of L_1_, M_5_, M_6_, M_8_, and S_9_ chromosomes (Fig. 1c, d).

**Figure 2.**
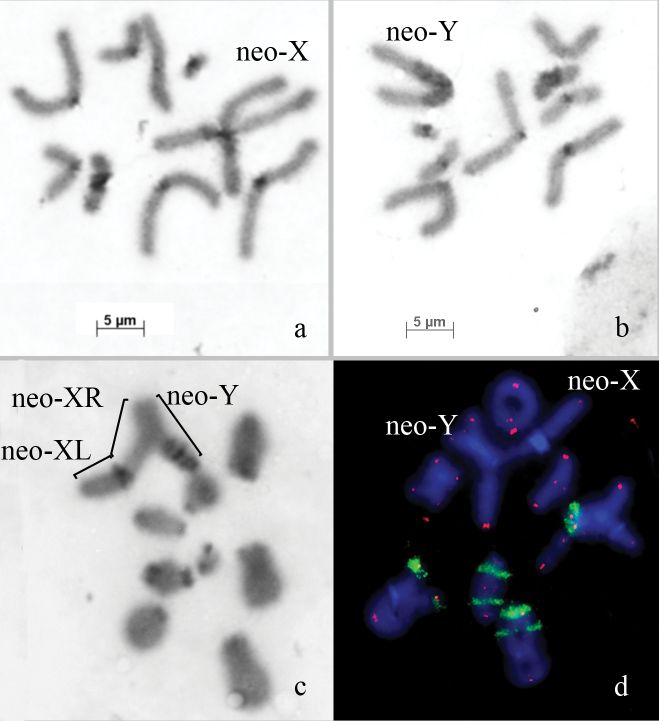
*Asiotmethis turritus* – **a** C-banded anaphase I chromosome with the neo-X chromosome **b** C-banded anaphase I chromosomes with the neo-Y chromosome **c** metaphase I chromosomes with C-band **d** FISH of 18S rDNA probe (green) and a telomeric repeat probe (red) with metaphase I chromosomes. Bar: 5 µm.

**Karyotype description of *Asiotmethis turritus* (Fischer von Waldheim, 1833).** The karyotype of *Asiotmethis turritus* consisted of 18 chromosomes (2n♂=18; 16AA+neo-X+neo-Y). Autosomes were acrocentric and fell into three groups according to their size: 3 large (L_1_–L_3_), 4 medium (M_4_–M_7_), and 1 small (S_8_) chromosome pair. The neo-X chromosome was submetacentric and the largest chromosome in the karyotype. The neo-Y chromosome was a large acrocentric, in size equal to the XR arm of the neo-X (Fig. 2a, b). Partial pairing in the prophase of meiosis and the formation of two chiasmata between the neo-Y and the XR indicated homology of the neo-Y and XR distal regions (Fig. 2c, d). Cheterochromatic blocks of different sizes are located in the pericentric regions of all autosomes (Fig. 2a ,b, c). The Y-chromosome has a small pericentric C-band and several interstitial C-bands in its proximal part (Fig. 2b, c).

The telomeric DNA probe hybridized on the termini of all chromosomes (Fig. 2d). ITSs were not observed on *Asiotmethis turritus* chromosomes.

FISH with 18S rDNA probe returned a signal on the C-positive pericentric region of three pairs of large autosomes. At least on one of them, the cluster of sequences homologous to 18S rDNA was polymorphic in size (Fig. 2d). Interstitial clusters of rDNA were revealed in the proximal part of the L_1_ chromosome and in the middle of the M_6_ chromosome. Both interstitial clusters of rDNA were localized in C-negative regions (Fig. 2d).

**Figure 3.**
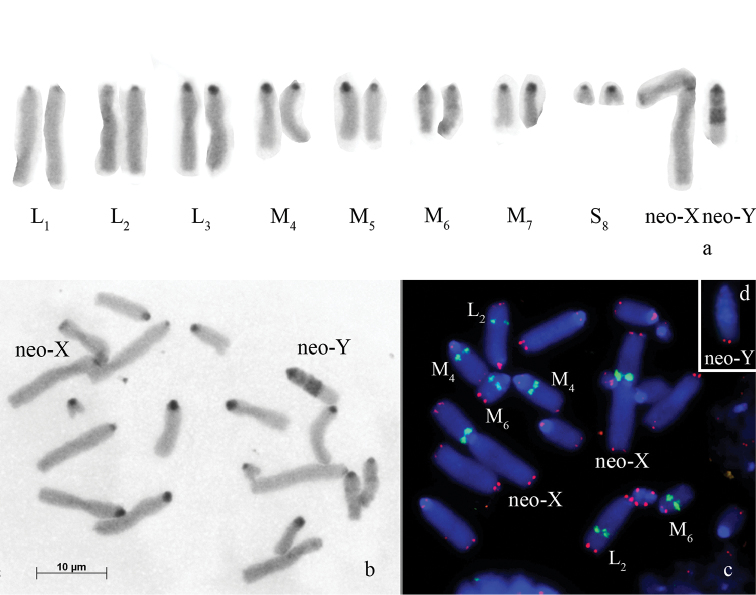
*Paranocaracris rubripes* – **a, b** C-banded mitotic chromosomes from embryos **c, d** FISH with 18S rDNA probe (green) and a telomeric repeat probe (red) on embryos mitotic metaphase chromosomes **c** female metaphase with two metacentric neo-X chromosomes **d** neo-Y chromosome. Bar: 10 µm.

**Karyotype description of *Paranocaracris rubripes* (Fischer von Waldheim, 1846).** The karyotype of *Paranocaracris rubripes* consisted of 18 chromosomes (2n♂=18; 16AA+neo-X+neo-Y). Autosomes were acrocentric and fell into three groups according to size: 3 large (L_1_–L_3_), 4 medium (M_4_–M_7_), and 1 small (S_8_) chromosome pair. The neo-X chromosome was here again submetacentric and the largest chromosome in the karyotype. The neo-Y was acrocentric, distinctly smaller in size than the XR arm of the neo-X (Fig. 3a, b). Cheterochromatic blocks of different sizes are located in the pericentric regions of all autosomes. Interstitial C-positive blocks were observed in the proximal region of the M_6_ chromosome pair, and the proximal region of the XL arm, but stained less intensively than the pericentromeric C-blocks. A minute telomeric C-positive block was observed in the XL arm. Part of the neo-Y chromosome was strongly heterochromatinized. In the neo-Y chromosome one small and two medium-sized interstitial blocks were observed in its proximal half, in addition to a medium-sized pericentric C-positive block (Fig. 3a, b).

The telomeric DNA probe hybridized on the termini of all chromosomes (Fig. 3c). ITSs were not observed on *Paranocaracris rubripes* chromosomes.

Clusters of 18S rDNA repeats were localized interstitially in three pairs of autosomes (a distal cluster on the L_2_, a proximal cluster on the M_4_ and a proximal cluster on the M_6_ chromosomes) and in the neo-X-chromosome. In the neo-X chromosome and M_6_ autosome, clusters of 18S rDNA repeats were localized in interstitial C-positive blocks (Fig. 3c).

**Figure 4.**
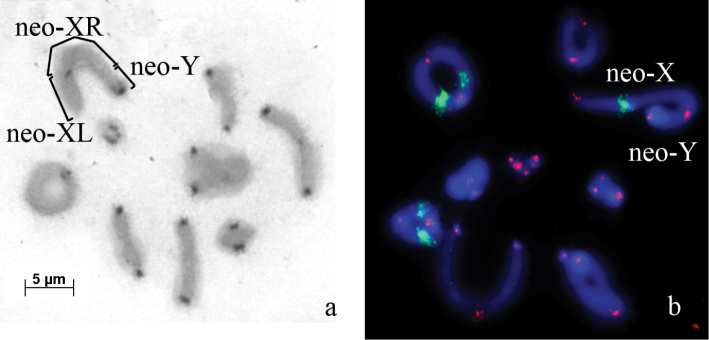
*Nocaracris cyanipes* – **a** C-banded meiotic metaphase I chromosomes **b** FISH with 18S rDNA probe (green) and a telomeric repeat probe (red) of meiotic metaphase I chromosomes. Bar: 5 µm.

**Karyotype description of *Nocaracris cyanipes* (Fischer von Waldheim, 1846**). The karyotype of *Nocaracris cyanipes* consisted of 18 chromosomes (2n♂=18; 16AA+neo-X+neo-Y). The karyotype and C-banding patterns of samples from the Armenian population proved to be similar to an earlier studied population from the North Caucasus (Bugrov and Warchałowska-Śliwa 1997). Autosomes were acrocentric and fell into three groups according to size: 3 large (L_1_–L_3_), 4 medium (M_4_–M_7_), and 1 small (S_8_) chromosome pair. The neo-X chromosome was again submetacentric and the largest chromosome in the karyotype. The neo-Y was acrocentric, distinctly smaller in size than the XR arm of the neo-X. Cheterochromatic blocks of medium size were located in the pericentric regions of all autosomes. An interstitial C-positive block was observed in the proximal region of the M_6_ chromosome pair, but it stained less intensively than the pericentromeric C-blocks. A small telomeric C-positive block was observed in the S_8_ chromosome. A minute telomeric C-positive block was observed in the XL arm, whereas the thin C-positive block on the M_6_ chromosomes described in samples from North Caucasian population was not found in the Armenian samples. Part of the neo-Y chromosome was strongly heterochromatinized. In the neo-Y chromosome, in addition to a pericentric C-block, a medium-sized interstitial block was observed in its proximal part. At meiotic prophase, large bivalents form two chiasmata, medium bivalents form 1 or 2 chiasmata, while small bivalents form only one chiasma. The neo-XY bivalent forms only one distal chiasma between the XR arm of the neo-X and the neo-Y (Fig. 4a, b).

The telomeric DNA probe hybridized on the termini of all chromosomes (Fig. 4b). ITSs were not observed on *Nocaracris cyanipes* chromosomes (Fig. 4b).

Clusters of 18S rDNA repeats were localized in two pairs of autosomes and in the neo-X chromosome. In the neo-X chromosome this cluster was localized in the proximal region of the XL arm. The autosomes carry clusters of rDNA in the interstitial regions of the L_3_ and M_6_ chromosomes (Fig. 4b).

**Figure 5.**
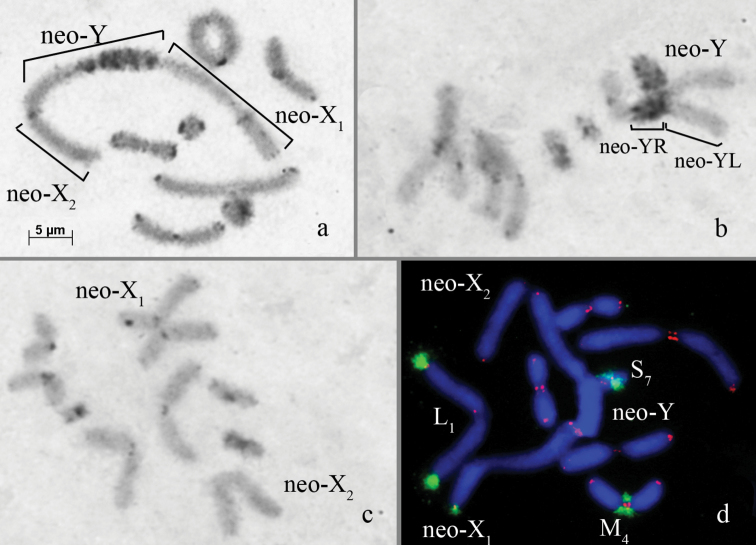
*Paranothrotes opacus* – **a** C-banded diakinesis chromosomes with the neo-X_1_, neo-X_2_ and neo-Y chromosomes **b** C-banded anaphase I chromosomes with the neo-Y chromosome **c** C-banded anaphase I chromosomes with the neo-Y chromosome **d** FISH of 18S rDNA probe (green) and a telomeric repeat probe (red) with diakinesis chromosomes. Bar: 5 µm.

**Karyotype description of *Paranothrotes opacus* (Brunner von Wattenwyl, 1882).** The male karyotype of *Paranothrotes opacus* consisted of 17 chromosomes (2n♂=14+X_1_X_2_Y.) Autosomes were acrocentric and fell into three groups according to size: 2 large (L_1_–L_2_), 4 medium (M_3_– M_6_), and 1 small (S_7_) chromosome pair. The neo-X_1_ and the neo-Y chromosomes were submetacentric and the largest chromosomes in the karyotype. The neo-X_2_ chromosome was one of the largest acrocentric chromosomes. Small-sized Cheterochromatic blocks were located in the pericentric regions of all autosomes and in the neo-X_1_. Minute terminal C-positive blocks were located on the L_1_, L_2_, M_3_, M_5_, M_6_, S_7_ chromosomes and both arms of the neo-X_1_ chromosome. An interstitial C-band was located in the proximal part of the X_1_R arm. The short arm (YL) of the Y-chromosome is strongly heterochromatinized with multiple small C-negative bands (Fig. 5a, b).

The telomeric DNA probe hybridized on the termini of all chromosomes (Fig. 5d). ITSs were not observed on *Paranothrotes opacus* chromosomes.

Clusters of 18S rDNA repeats were localized in the telomeric regions of the L_1_, M_4_, S_7_ chromosomes and the X_1_R arm of the X_1_ chromosome (Fig. 5d).

## Discussion

Comparative cytogenetic analysis of all the Armenian Pamphagidae grasshopper species studied showed karyotypes unusual for the family. The chromosome number previously considered standard for Pamphagidae grasshoppers (19 for males and 20 for females; (XO♂/XX♀) was found in only in one of the species studied, *Eremopeza festiva*. Nevertheless, the chromosome morphology in this species appeared to be different from the standard karyotype. In contrast to the acrocentric chromosomes of earlier studied species, all chromosomes in *Eremopeza festiva* were biarmed with short second arms. The formation of a biarmed chromosome from an initially acrocentric chromosome without changing the chromosome number in the karyotype is usually considered a result of pericentric inversions (White 1973, Hewitt 1979). Chromosome reorganization of this type has been described in many taxa of grasshoppers (Hewitt 1979), but in Pamphagidae similar chromosome evolution has only been discovered in *Melanotmethis fuscipennis*, with three pairs of autosomes and the X chromosome showing a short euchromatic arm (Bugrov and Warchałowska-Śliwa 1997). It is impossible to precisely identify the mechanism of such short arm formation, which could potentially be the result of chromosome region inversion or of centromere transposition by neocentromere formation. The ITSs discovered in pericentric C-blocks of four pairs of autosomes and the X could be considered an argument in favour of an explanation involving chromosome region inversion. We suppose that DNA amplification took part in the formation of at least some of the short arms. Repeats homologous to 18S rDNA enriched some of the short arms. DNA amplification involving rDNA also took place in the formation of C-positive blocks in the distal part of the long arms of the M_8_ and S_9_ chromosomes. It is should be noted that usually rDNA amplification or enrichment of some chromosome regions by rDNA leads to its heterochromatization (Bugrov et al. 2003, Jetybayev et al. 2012). We observed this phenomenon in the distal part of the long arms of the M_8_ and S_9_ chromosomes but short arms of the chromosomes remained C-negative. Their DNA content is now a question of special interest and can be revealed by sequencing the microdissected material of the short arms (Bugrov et al. 2007).

In three of the species studied (*Asiotmethis turritus*, *Nocaracris cyanipes*, *Paranocaracris rubripes*), the autosomes looked like standard autosomes for Pamphagidae grasshoppers except those autosomes involved in translocation with the X chromosomes. In all three of these species, the chromosome number was 2n=18 in both males and females and the karyotype included sex chromosomes untypical for grasshoppers: neo-XY♂/neo-XX♀. Furthermore, whereas the autosomes showed conservatism we observed intensive evolution in the sex chromosomes. Obviously, the first step of neo-X and neo-Y formation was a translocation between an ancient acrocentric X chromosome and a large acrocentric autosome. In *Asiotmethis turritus* we observed the next step of sex chromosome evolution: the neo-Y acquired small interstitial C-positive blocks in its proximal region. As a result, we observed heteromorphization of initial homologues elements, namely XR arms of the neo-X and neo-Y chromosomes. In prophase I of meiosis the XR and neo-Y were similar in length, conjugated with their distal C-negative regions, and formed a bivalent, usually with two chiasmata. The proximal region of the neo-Y chromosome, which was enriched with repeated sequences, was not involved in pairing and recombination (Fig. 2). In *Nocaracris cyanipes* and *Paranocaracris rubripes*, on the other hand, we probably observed the neo-Y chromosome after yet another next step of evolution: the neo-Y chromosome in these species was significantly shorter in comparison with the XR and showed a significantly larger heterochromatic part than was observed in the neo-Y chromosome of *Asiotmethis turritus*. Regardless of the form of autosomal bivalents observed in the species studied, XR and the neo-Y chromosome in *Nocaracris cyanipes*, *Paranocaracris rubripes* were associated only in the distal regions. A similar type of pairing was previously reported for species of the Nocarodeini tribe (Bugrov and Warchałowska-Śliwa 1997, Bugrov and Grozeva 1998).

In *Paranothrotes opacus* we discovered a new chromosome sex determination system for Pamphagidae grasshoppers (neo-X_1_X_2_Y♂/neo-X_1_X_1_X_2_X_2_♀), which reflects the most advanced stage of sex chromosome evolution in the Nocarodeini tribe. This form of sex determination was the result of an additional reciprocal translocation involving the medium C-positive neo-Y-chromosome and a large autosome. As such, this chromosome reorganization might be considered the next step of sex chromosome evolution in comparison with *Asiotmethis turritus*, *Nocaracris cyanipes*, *Paranocaracris rubripes*, and species belong to the tribe Nocarodeini tribe. This led to the transformation of a typical Nocarodeini acrocentric neo-Y chromosome into a submetacentric neo-Y-chromosome. In this species, the unpaired acrocentric autosome becomes another heterosome – a neo-X_2_ chromosome. This finding emphasizes once again the promising prospects for studying the Pamphagidae karyotype as a research model of sex chromosome evolution.

FISH using 18S rDNA with chromosomes of Pamphagidae grasshoppers showed that the clusters of rDNA differ in size and location among the species studied. In *Eremopeza festiva* they were found in the pericentromeric C-positive regions of all chromosomes, but could be different in size even on homologous chromosomes. In other species, rDNA clusters are usually localized in the pericentromeric, intercalary or telomeric region of two, three or four pairs of chromosomes, including the neo-X chromosome. It should be emphasized that in *Asiotmethis turritus* one pair of autosomes showed double rDNA clusters. Three clusters of rDNA on one chromosome were previously reported for *Pamphagus ortolaniae* (Vitturi et al. 2008). Multiple localizations of rDNA clusters in a chromosome is a very rare type of rDNA cluster distribution (Cabrero and Camacho 2008, Jetybayev et al. 2012, Palacios-Gimenez et al. 2013).

In most of the species studied, the clusters of telomeric repeats were located in chromosome termini. However, some chromosomes of *Eremopeza festiva* showed ITSs (Fig. 1e). One of the possible sources of ITSs might be pericentric inversions, which led to the formation of biarmed chromosomes. However, ITSs were not revealed in the regions of chromosome fusion leading to neo-X, chromosome of *Asiotmethis turritus*, *Nocaracris cyanipes*, *Paranocaracris rubripes* and neo-X_1_, neo-Y chromosomes in *Paranothrotes opacus*. These findings suggest that translocation of the X-chromosome and autosome was accompanied with the loss of small regions containing telomeric repeats.

## Conclusion

The karyotype of Pamphagidae grasshoppers was once considered to be among the very conservative (Camacho et al. 1981). However, we have found Armenian Pamphagidae grasshopper species to be characterized by intensive karyotypic evolution.

Overall, two different types of karyotype reorganization were found to be evidenced in Armenian Pamphagidae grasshoppers. In *Eremopeza festiva*, evolutionary chromosome rearrangements have led to a karyotype consisting of exclusively biarmed chromosomes with numerous C-positive regions enriched with repeats homologous to rDNA. We suppose that one of the important evolutionary processes that led to the formation of *Eremopeza festiva*’s modern biarmed karyotype involved the massive amplification of repetitive DNA.

The karyotypes of *Asiotmethis turritus*, *Nocaracris cyanipes* and *Paranocaracris rubripes*, in turn, were formed as a result of translocations involving the X chromosome and one of the autosomes. Furthermore, neo sex chromosomes showed additional evolutionary changes, namely, the formation of C-positive regions and the loss of euchromatic regions. Comparison of the sex chromosomes in these species revealed different stages of Y chromosome evolution.

The classical model of sex chromosome evolution postulates that sex chromosome degradation takes place due to suppression of recombination between parts of the sex chromosomes; in evolution the region of one of the sex chromosomes accumulates repetitive sequences and loses euchromatic gene-rich material. These processes lead to heterochromatinization and shrinking of one of the sex chromosome (Muller 1914, White 1973, Charlesworth et al. 2005, Traut et al. 2008). The neo-Y chromosomes of Pamphagidae grasshoppers have gone through the typical Y chromosome evolutionary stages, which could be identified in the species studied. Initial accumulation of C-positive blocks was observed in the neo-Y chromosome of *Asiotmethis turittus*, whereas the neo-Y chromosome of *Nocaracris cyanipes* and *Paranocaracris rubripes* showed an advanced stage of sex chromosome evolution associated with the loss of the part of C-negative regions. In *Paranothrotes opacus*, the neo-Y chromosome have gone through a more complex rearrangement: a partial heterochromatization and fusion with an autosome, generating a complex system of sex chromosomes, (neo-X_1_X_2_Y♂/neo-X_1_X_1_X_2_X_2_♀).

Taken together, the different sets of sex chromosomes in the Armenian Pamphaginae species studied provide evidence that the Pamphagidae family offers an excellent model for studying the mechanisms of sex chromosome evolution. Its advantage lies in the availability of different stages of sex chromosome evolution, ranging from an initial XO♂/XX♀ sex determination system to a newly arisen neo-XY♂/XX♀ system, an advanced neo-XY♂/XX♀ system with significantly degraded neo-Y chromosome and even a very complicated neo-X_1_X_2_Y♂/ neo-X_1_X_1_X_2_X_2_♀ sex determination system.
